# Multi-muscle deep learning segmentation to automate the quantification of muscle fat infiltration in cervical spine conditions

**DOI:** 10.1038/s41598-021-95972-x

**Published:** 2021-08-16

**Authors:** Kenneth A. Weber, Rebecca Abbott, Vivie Bojilov, Andrew C. Smith, Marie Wasielewski, Trevor J. Hastie, Todd B. Parrish, Sean Mackey, James M. Elliott

**Affiliations:** 1 Northern Sydney Local Health District, The Kolling Institute, St. Leonards, NSW Australia; 2 The Faculty of Medicine and Health, The University of Sydney, Camperdown, NSW Australia; 3 Division of Pain Medicine, Department of Anesthesiology, Perioperative and Pain Medicine, Stanford University School of Medicine, Palo Alto, CA USA; 4 Department of Physical Therapy and Human Movement Sciences, Feinberg School of Medicine, Northwestern University, Chicago, IL USA; 5 Physical Therapy Program, Department of Physical Medicine and Rehabilitation, School of Medicine, University of Colorado, Aurora, CO USA; 6 Statistics Department, Stanford University, Palo Alto, CA USA; 7 Department of Radiology, Northwestern University, Chicago, IL USA

**Keywords:** Musculoskeletal system, Magnetic resonance imaging, Biomarkers

## Abstract

Muscle fat infiltration (MFI) has been widely reported across cervical spine disorders. The quantification of MFI requires time-consuming and rater-dependent manual segmentation techniques. A convolutional neural network (CNN) model was trained to segment seven cervical spine muscle groups (left and right muscles segmented separately, 14 muscles total) from Dixon MRI scans (n = 17, 17 scans < 2 weeks post motor vehicle collision (MVC), and 17 scans 12 months post MVC). The CNN MFI measures demonstrated high test reliability and accuracy in an independent testing dataset (n = 18, 9 scans < 2 weeks post MVC, and 9 scans 12 months post MVC). Using the CNN in 84 participants with scans < 2 weeks post MVC (61 females, 23 males, age = 34.2 ± 10.7 years) differences in MFI between the muscle groups and relationships between MFI and sex, age, and body mass index (BMI) were explored. Averaging across all muscles, females had significantly higher MFI than males (*p* = 0.026). The deep cervical muscles demonstrated significantly greater MFI than the more superficial muscles (*p* < 0.001), and only MFI within the deep cervical muscles was moderately correlated to age (r > 0.300, *p* ≤ 0.001). CNN’s allow for the accurate and rapid, quantitative assessment of the composition of the architecturally complex muscles traversing the cervical spine. Acknowledging the wider reports of MFI in cervical spine disorders and the time required to manually segment the individual muscles, this CNN may have diagnostic, prognostic, and predictive value in disorders of the cervical spine.

## Introduction

Spinal conditions are a leading cause of pain and physical disability world-wide, afflicting an estimated one billion people across the globe^1–4^. Conventional medical imaging (radiographs, computed tomography (CT), and magnetic resonance imaging (MRI)) provides excellent visualization of the spinal anatomy and pathology. However, spinal conditions often present with multi-level pathological changes^5^, and incidental findings are frequently present even in asymptomatic individuals^6^, questioning the predictive value and clinical relevance of conventional imaging for long-term outcomes^7–10^. The recent application of artificial intelligence methods (e.g. convolutional neural networks (CNNs)) to image analysis is transforming medical imaging by providing the power to efficiently and automatically extract many quantitative metrics from images not previously possible in the typical clinical workflow^11^. Using data-driven approaches, these complementary imaging metrics, combined with examination and imaging findings, may lead to an improved understanding of spinal disease and deliver more sensitive and specific measures of spinal pathology with greater diagnostic, prognostic, and predictive value^12,13^. Artificial intelligence combined with precision medicine approaches can enable augmented intelligence-driven medical decision making and the personalized delivery of health care^14^.

One example is the infiltration of the spinal musculature with fat, muscle fat infiltration (MFI), which has been consistently observed in patients with cervical spine conditions, including degenerative cervical myelopathy, traumatic spinal cord injury, and whiplash from a motor vehicle collision (MVC)^15–17^. MFI appears to be uniquely present in those with poor functional recovery, suggesting its presence may represent a modifiable target for treatment. While the pathophysiological and pathomechanical mechanisms underlying these spinal conditions differ, the characteristic pattern of MFI appears to occur with greatest magnitude in the deep muscular layers of the cervical extensors (i.e., multifidus and semispinalis cervicis (MFSS)) and cervical flexors (i.e. longus colli and longus capitis (LC))^18–20^. The ubiquitous nature of muscle compositional changes in these spinal conditions suggests MFI may be one common biological explanation or risk factor for persisting neck-related disability. The magnitude of MFI could have clinical implications for the management of the spine and recovery from persistent spinal disorders. For example, new evidence suggests that higher pre-surgical MFI of the LC in degenerative cervical myelopathy is associated with reduced post-surgical improvement in physical function^21^.

Using manual methods, MFI can be quantified from conventional (T_1_- and T_2_-weighted) and advanced (Dixon and proton density fat fraction) MRI and CT^20,22,23^. Manual segmentation of spinal muscles is not efficiently performed in a clinical environment, thereby limiting its use to research environments. Recently, we trained a deep-learning CNN model to perform segmentation of a single cervical spine muscle group (i.e., MFSS) in participants with varying levels of whiplash-related pain and disability following a MVC^24^. We reported high accuracy and reliability of the CNN MFI measures in an independent testing dataset and demonstrated higher MFSS MFI in patients with persisting pain and neck-related disability at 3 months post MVC versus those participants nominating full recovery. The CNN markedly improved the efficiency of the segmentation, reducing the processing time from 20 min per image to only seconds. In addition to our work, CNN’s have been used to automate the segmentation of the lumbar paraspinal and iliopsoas muscles from T_1_-weighted and Dixon MRI scans, respectively^25,26^. CNN’s have also been applied to other spinal structures, allowing for the automatic quantification of vertebrae and intervertebral disc morphology from MRI^27^.

Other cervical spine muscle groups, in addition to the MFSS, have been implicated in cervical spine conditions, and the assessment of multiple muscles is desired for a comprehensive evaluation of cervical spine muscle composition^16^. The time required for manual segmentation scales with the number and architectural complexity of the muscle groups being segmented, providing the motivation for developing accurate and reliable automated multi-muscle segmentation methods to be used beyond the research environment with a target for clinical implementation. Here, we trained and tested a CNN to segment seven cervical spine muscles groups (left and right muscles segmented separately): MFSS, LC, semispinalis capitis (SSCap), splenius capitis (SPCap), levator scapula (LS), sternocleidomastoid (SCM), and trapezius (TR) using high-resolution Dixon fat-water MRI (Table 1). We then explored relationships between the CNN-derived MFI measures and sex and age, known risk factors for chronic whiplash symptoms, as well as body mass index (BMI) in participants with a whiplash injury imaged within two weeks of a MVC. We hypothesized that higher levels of MFI would be associated with older age, female sex, and higher BMI^28–30^.

**Table 1:** Muscle abbreviations.

Muscle	Abbreviation	Vertebral levels	Label (Left/Right)
Multifidus and Semispinalis Cervicis	MFSS	C4–C6	2/1
Longus Colli and Longus Capitis	LC	C3–C6	4/3
Semispinalis Capitis	SSCap	C3–C5	8/7
Splenius Capitis	SPCap	C3–C5	6/5
Levator Scapula	LS	C5–C6	14/13
Sternocleidomastoid	SCM	C4–C6	10/9
Trapezius	TR	C6	12/11

## Results

### CNN accuracy and reliability

**Figure 1:**
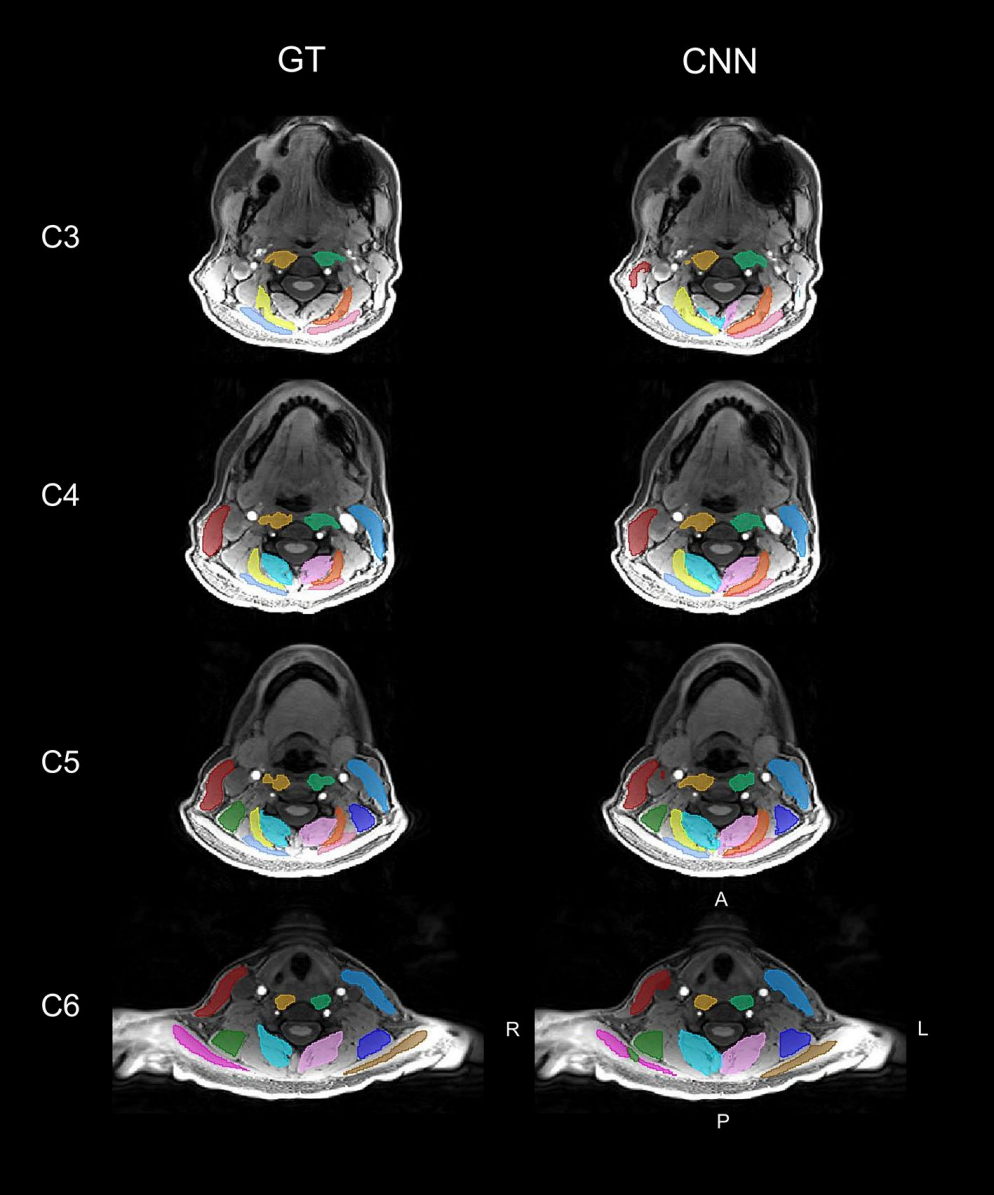
Multi-muscle convolutional neural network (CNN) segmentation of seven cervical spine muscle groups. CNN segmentation masks for the ground truth (GT) and CNN from a randomly selected testing scan are shown overlaid the water-only image. Axial images at the C3–C6 vertebral levels were selected to show changes in muscle morphometry across the cervical spine. The muscle groups segmented included the MFSS (left = light pink, right = aqua), LC (left = light green, right = gold), SSCap (left = orange, right = yellow), SPCap (left = dark pink, right = light blue), LS (left = indigo, right = dark green), SCM (left = blue, right = red), and TR (left = brown, right = magenta). The artifact in the left jaw is due to metal from dental work. See Fig. 2 for three-dimensional renderings of the GT and CNN segmentations. L = left, R = right, A = anterior, P = posterior.

**Figure 2:**
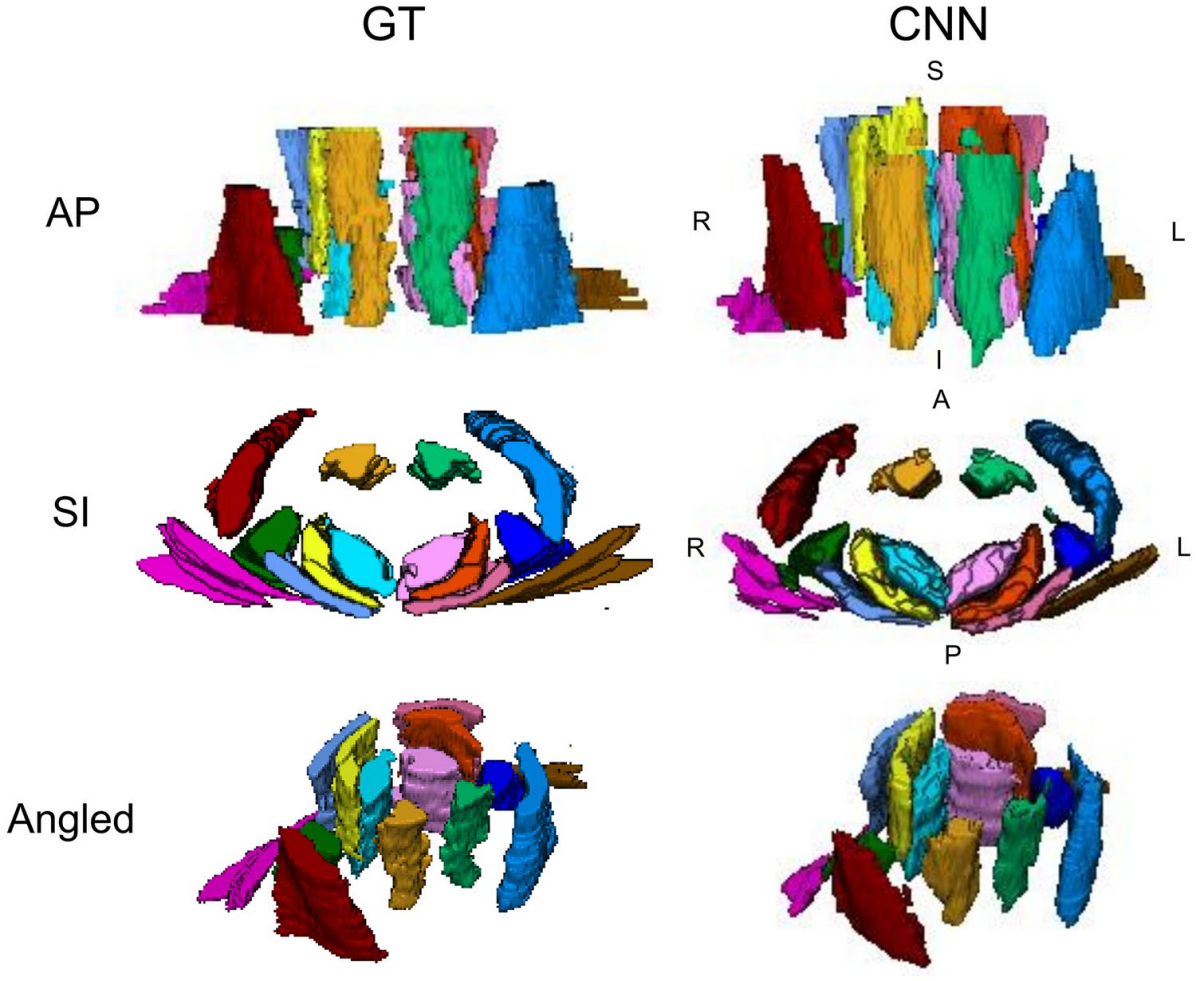
Three-dimensional renderings of the cervical spine muscle segmentations. The ground truth (GT) and convolutional neural network (CNN) segmentations from a randomly selected testing scan are shown as three-dimensional renderings. The muscle groups segmented included the MFSS (left = light pink, right = aqua), LC (left = light green, right = gold), SSCap (left = orange, right = yellow), SPCap (left = dark pink, right = light blue), LS (left = indigo, right = dark green), SCM (left = blue, right = red), and TR (left = brown, right = magenta). See Fig. 1 for the two-dimensional segmentation masks of the GT and CNN overlaid the water-only image. L = left, R = right, A = anterior, P = posterior, S = superior, I = inferior.

**Figure 3:**
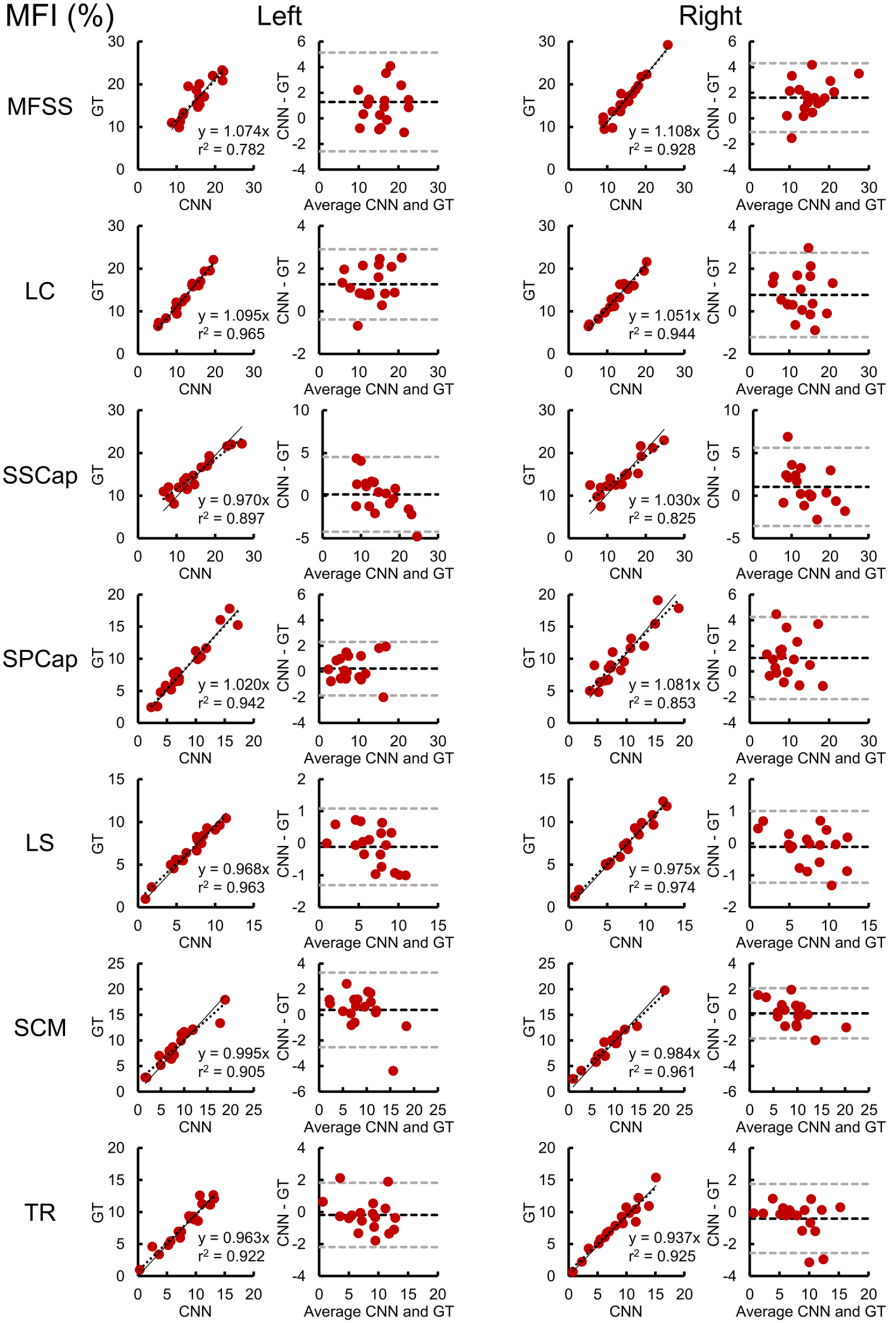
Reliability and accuracy of the convolutional neural network (CNN) muscle fat infiltration (MFI) measures on the testing dataset (n = 18). Correlation and Bland-Altman plots are shown for MFI for each of the muscle groups. In the correlation plots, the dashed black line represents the best fit line, and the linear regression coefficient (β) of the ground truth (GT) on CNN (intercept = 0) is also provided (solid black line), which can be used to correct the CNN measurement bias. In the Bland-Altman plots, the dashed black and gray lines indicate the mean difference (i.e., bias) ± 1.96 × standard deviation (i.e., 95% limits of agreement). See Table 3 for the intraclass correlation coefficients (ICC_2,1_).

**Figure 4:**
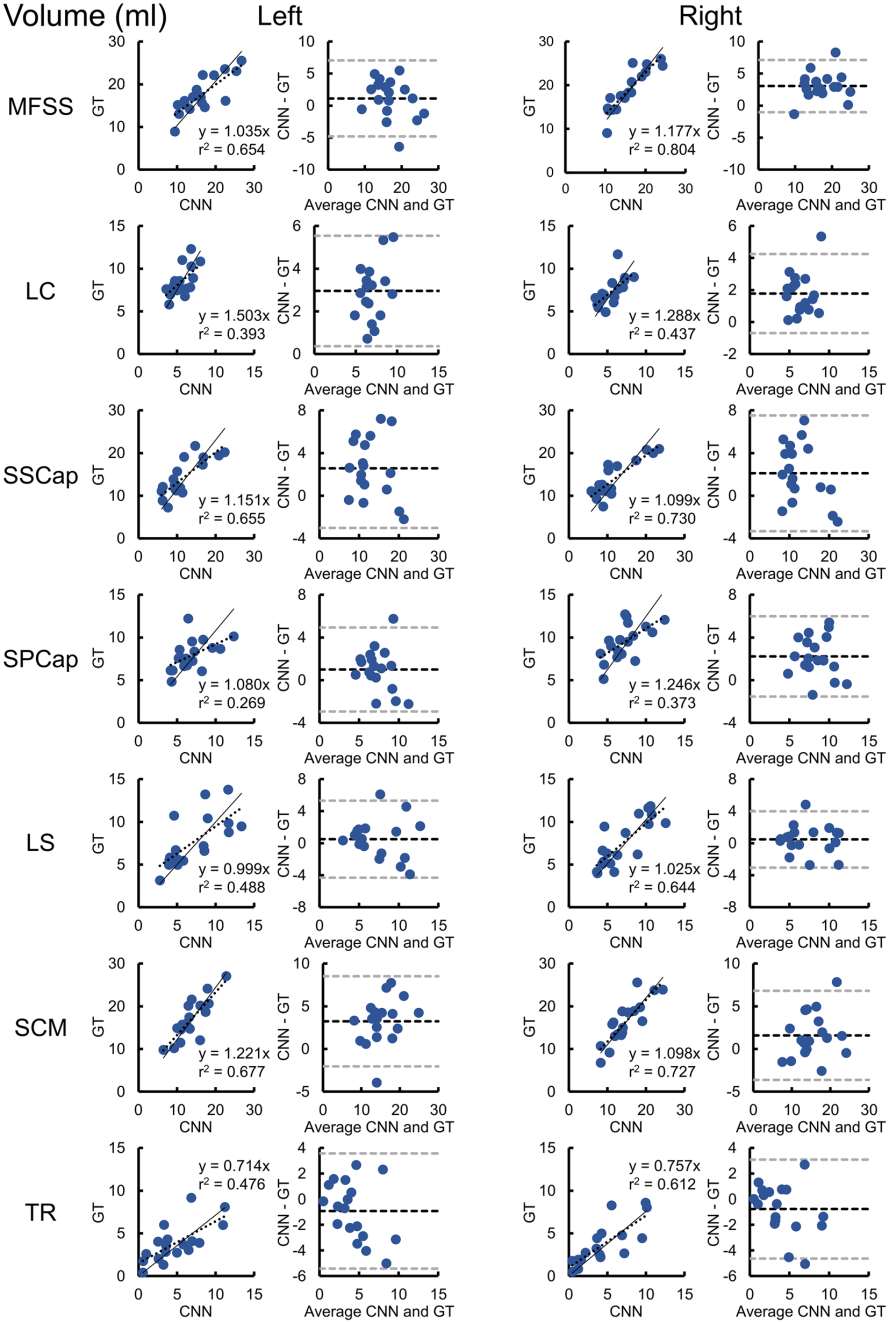
Reliability and accuracy of the convolutional neural network (CNN) muscle volumes on the testing dataset (n = 18). Correlation and Bland-Altman plots are shown for volume for each of the muscle groups. In the correlation plots, the dashed black line represents the best fit line, and the linear regression coefficient (β) of the ground truth (GT) on CNN (intercept = 0) is also provided (solid black line), which can be used to correct the CNN measurement bias. In the Bland-Altman plots, the dashed black and gray lines indicate the mean difference (i.e., bias) ± 1.96 × standard deviation (i.e., 95% limits of agreement). See Table 3 for the intraclass correlation coefficients (ICC_2,1_).

Training of the CNN segmentation model was completed in 100,000 iterations (Supplementary Material Fig. 1), and the accuracy and reliability of the trained CNN model was evaluated on the independent testing dataset (n = 18). Figures 1 and 2 compare the GT segmentations to the CNN segmentations from a randomly selected testing scan. The CNN accuracy for the primary measure of MFI was high; for all muscle groups, the absolute value of the mean bias in MFI was less than 2.0%, the MFI mean absolute error (MAE) was less than 2.0%, and the MFI root mean squared error (RMSE) was less than 3.0% (Table 2 and Fig. 3). Likewise, the reliability of the CNN MFI measures was excellent with the ICC_2__,__1_ exceeding 0.800 for all muscle groups (Table 3 and Fig. 3)^31^. The accuracy and reliability of the secondary measure of muscle volume were generally lower than the MFI measurements (Table 2, 3, and Fig. 4). While the reliability of the MFSS, SSCap, LS, SCM, and TR muscle volume was good (ICC_2,1_ > 0.600), the reliability of the SPCap and LC volumes were fair (ICC_2,1_ = 0.407–0.462) to poor (ICC_2,1_ = 0.207–0.395), respectively (Table 3 and Fig. 4). The mean Sørensen-Dice index between the CNN and GT was > 0.65 for all muscle groups except the TR (Sørensen-Dice index < 0.50). The volume ratios were greater than one for all muscles indicating that the CNN model generated segmentations of a larger volume than the GT. See Table 4 for a summary of all segmentation metrics.

**Table 2:** CNN MFI and volume accuracy.

Muscle	MFI (%)						Volume (ml)					
	Mean	Bias	95% LA	MAE	RMSE	r^2^	Mean	Bias	95% LA	MAE	RMSE	r^2^
**MFSS**												
Left	16.8 ± 1.0	1.3	−2.6–5.1	1.7	2.3	0.782	17.5 ± 1.0	1.1	−4.8–7.1	2.7	3.2	0.654
Right	16.4 ± 1.2	1.6	−1.1–4.3	1.8	2.1	0.928	18.8 ± 1.1	3.0	−1.0–7.1	3.2	3.7	0.804
**LC**												
Left	13.7 ± 1.0	1.3	−0.4–2.9	1.3	1.5	0.965	8.4 ± 0.4	3.0	0.4–5.5	3..0	3.2	0.393
Right	13.3 ± 1.0	0.8	−1.2–2.7	1.0	1.2	0.944	7.3 ± 0.4	1.8	−0.7–4.3	1.8	2.2	0.437
**SSCap**												
Left	14.9 ± 1.0	0.1	−4.2–4.5	1.7	2.2	0.897	14.2 ± 1.0	2.6	−3.0–8.2	3.1	3.8	0.655
Right	14.5 ± 1.0	1.0	−3.6–5.6	1.9	2.5	0.825	13.9 ± 1.0	2.1	−3.3–7.5	2.8	3.4	0.730
**SPCap**												
Left	8.8 ± 1.0	0.2	−1.8–2.3	0.9	1.1	0.942	7.9 ± 0.4	1.0	−2.9–4.9	1.8	2.2	0.269
Right	10.1 ± 1.0	1.1	−2.1–4.3	1.5	1.9	0.853	9.5 ± 0.5	2.2	−1.5–6.0	2.4	2.9	0.373
**LS**												
Left	6.7 ± 0.6	−0.1	−1.3–1.1	0.5	0.6	0.963	7.6 ± 0.7	0.5	−4.3–5.3	1.9	2.4	0.488
Right	7.4 ± 0.7	−0.1	−1.2–1.0	0.4	0.6	0.974	7.6 ± 0.7	0.5	−3.1–4.0	1.4	1.8	0.644
**SCM**												
Left	9.0 ± 0.9	0.4	−2.5–3.3	1.1	1.5	0.905	16.9 ± 1.1	3.2	−2.0–8.5	3.7	4.2	0.677
Right	9.2 ± 0.9	0.1	−1.8–2.1	0.8	1.0	0.961	16.3 ± 1.2	1.6	−3.6–6.8	2.3	3.0	0.727
**TR**												
Left	8.0 ± 0.8	−0.2	−2.2–1.8	0.8	1.0	0.922	3.9 ± 0.5	−0.9	−5.4–3.6	2.0	2.4	0.476
Right	7.8 ± 0.8	−0.4	−2.6–1.7	0.7	1.1	0.925	3.7 ± 0.6	−0.8	−4.6–3.1	1.6	2.1	0.612

Bland-Altman plots and Pearson correlations were used to assess the accuracy of the convolutional neural network (CNN) model compared to the ground truth (GT) for muscle fat infiltration (MFI) and muscle volume measures on the testing dataset (n = 18) (Fig. 3, 4). Mean = mean CNN measure ± 1 standard error. Bias = mean difference between CNN and GT. LA = limits of agreement. MAE = mean absolute error. RMSE = root mean squared error.

**Table 3:** CNN MFI and volume reliability.

Muscle	MFI (%)			Volume (ml)		
	ICC_2,1_	95% CI	*p*	ICC_2,1_	95% CI	*p*
**MFSS**						
Left	0.847	0.541–0.946	< 0.001	0.781	0.510–0.911	< 0.001
Right	0.902	0.280–0.974	< 0.001	0.733	−0.056–0.927	< 0.001
**LC**						
Left	0.941	0.214–0.987	< 0.001	0.207	−0.074–0.583	0.003
Right	0.957	0.804–0.987	< 0.001	0.395	−0.112–0.751	0.001
**SSCap**						
Left	0.910	0.777–0.966	< 0.001	0.699	0.129–0.895	< 0.001
Right	0.870	0.675–0.950	< 0.001	0.765	0.313–0.917	< 0.001
**SPCap**						
Left	0.971	0.925–0.989	< 0.001	0.462	0.044–0.752	0.014
Right	0.898	0.668–0.965	< 0.001	0.407	−0.099–0.747	0.003
**LS**						
Left	0.975	0.934–0.990	< 0.001	0.699	0.363–0.875	< 0.001
Right	0.985	0.960–0.994	< 0.001	0.800	0.550–0.919	< 0.001
**SCM**						
Left	0.937	0.844–0.976	< 0.001	0.635	−0.047–0.881	< 0.001
Right	0.972	0.927–0.989	< 0.001	0.806	0.489–0.928	< 0.001
**TR**						
Left	0.958	0.893–0.984	< 0.001	0.629	0.258–0.841	0.001
Right	0.954	0.882–0.983	< 0.001	0.742	0.438–0.894	< 0.001

Intraclass correlation coefficients (ICC_2,1_, two-way random, absolute agreement, single measure) were used to assess the reliability of the convolutional neural network (CNN) model compared to the ground truth (GT) for the muscle fat infiltration (MFI) and muscle volume measures on the testing dataset (n = 18). CI = confidence interval. *p* = F-test with true value of 0.

**Table 4:** CNN segmentation performance metrics.

Muscle	Dice	JI	CC	TPR	TNR	PPV	VR
**MFSS**							
Left	0.79 ± 0.01	0.65 ± 0.01	0.45 ± 0.04	0.83 ± 0.02	1.00 ± 0.00	0.76 ± 0.02	1.10 ± 0.05
Right	0.78 ± 0.01	0.64 ± 0.01	0.44 ± 0.02	0.86 ± 0.01	1.00 ± 0.00	0.72 ± 0.01	1.21 ± 0.04
**LC**							
Left	0.70 ± 0.01	0.54 ± 0.02	0.11 ± 0.05	0.89 ± 0.02	1.00 ± 0.00	0.58 ± 0.02	1.58 ± 0.07
Right	0.71 ± 0.01	0.56 ± 0.01	0.18 ± 0.05	0.84 ± 0.02	1.00 ± 0.00	0.63 ± 0.02	1.36 ± 0.06
**SSCap**							
Left	0.73 ± 0.01	0.58 ± 0.02	0.24 ± 0.05	0.83 ± 0.02	1.00 ± 0.00	0.66 ± 0.02	1.29 ± 0.07
Right	0.74 ± 0.01	0.59 ± 0.02	0.28 ± 0.05	0.83 ± 0.02	1.00 ± 0.00	0.68 ± 0.03	1.26 ± 0.07
**SPCap**							
Left	0.68 ± 0.02	0.52 ± 0.02	0.02 ± 0.08	0.75 ± 0.03	1.00 ± 0.00	0.64 ± 0.02	1.20 ± 0.07
Right	0.67 ± 0.01	0.51 ± 0.01	0.01 ± 0.05	0.79 ± 0.02	1.00 ± 0.00	0.60 ± 0.02	1.37 ± 0.08
**LS**							
Left	0.76 ± 0.01	0.62 ± 0.02	0.36 ± 0.04	0.86 ± 0.02	1.00 ± 0.00	0.69 ± 0.02	1.26 ± 0.05
Right	0.76 ± 0.01	0.62 ± 0.02	0.36 ± 0.04	0.80 ± 0.02	1.00 ± 0.00	0.73 ± 0.02	1.11 ± 0.05
**SCM**							
Left	0.73 ± 0.02	0.58 ± 0.02	0.20 ± 0.11	0.77 ± 0.03	1.00 ± 0.00	0.71 ± 0.03	1.15 ± 0.09
Right	0.74 ± 0.02	0.59 ± 0.02	0.26 ± 0.08	0.78 ± 0.03	1.00 ± 0.00	0.72 ± 0.03	1.12 ± 0.07
**TR**							
Left	0.41 ± 0.04	0.27 ± 0.03	−2.01 ± 0.40	0.49 ± 0.06	0.94 ± 0.06	0.46 ± 0.03	1.09 ± 0.17
Right	0.45 ± 0.04	0.30 ± 0.03	−2.40 ± 0.83	0.46 ± 0.05	1.00 ± 0.00	0.50 ± 0.05	1.07 ± 0.18

The convolutional neural network (CNN) segmentation performance was further assessed on the testing dataset (n = 18) using the Sørensen-Dice index (Dice), Jaccard index (JI), conformity coefficient (CC), true positive rate (TPR), true negative rate (TNR), positive predictive value (PPV), and volume ratio (VR). Metrics shown = mean ± 1 standard error.

### Interrater reliability

Another trained independent rater segmented a subset of the dataset (n = 10) to assess the reliability of the manual segmentations between human raters. Interrater reliability for the MFI measures was excellent (ICC_2__,__1_ > 0.800) for most muscles except for the left SSCap, which demonstrated good interrater reliability (ICC_2,1_ = 0.742) (Supplementary Table 2). Similarly, the interrater reliability of the muscle volume measures was excellent (ICC_2__﻿,__1_ > 0.800) for most muscle groups with only a few exceptions. The left and right LC and right LS demonstrated good interrater reliability (ICC_2,1_ > 0.600). The left and right TR muscle, however, demonstrated poor interrater reliability (ICC_2__,__1_ < 0.300) (Supplementary Table 2, Supplementary Figs. 2,  3).

### MFI characterization

**Figure 5:**
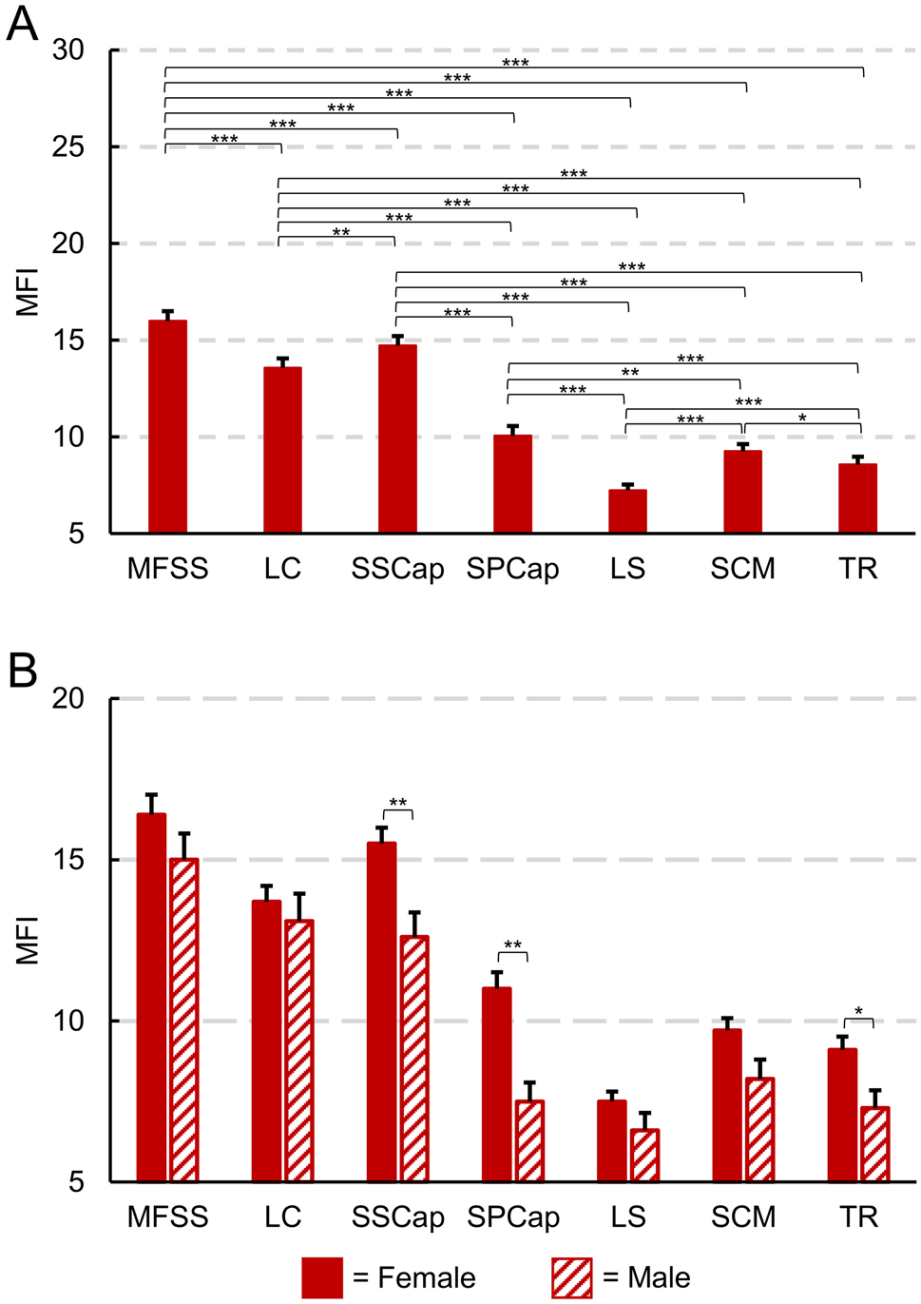
Muscle fat infiltration (MFI) of each muscle was calculated from the convolutional neural network (CNN) segmentations in 84 participants from the first time point (< 2 weeks following a motor vehicle collision, 61 females, 23 males, age = 34.2 ± 10.7 years). The left and right MFI measures for each muscle group were averaged for these analyses. (**A**) Between muscle group comparison of MFI. Each muscle group had significantly different MFI (paired t-tests) with the deep cervical extensors and flexors having higher MFI than the more superficial layers. (**B**) MFI by sex for each muscle group. When averaging across all muscle groups, female participants had significantly greater MFI than male participants (1.8% ± 0.8% higher MFI in females, *p* = 0.026). Females had significantly higher MFI than males in the SSCap, SPCap, and TR (one-way ANCOVA controlling for age and BMI). Estimated marginal means are shown. See Table 2 and Supplementary Table 3 for additional information. Error bars = 1 standard error. *p < 0.05, **p < 0.01, ***p < 0.001.

**Figure 6:**
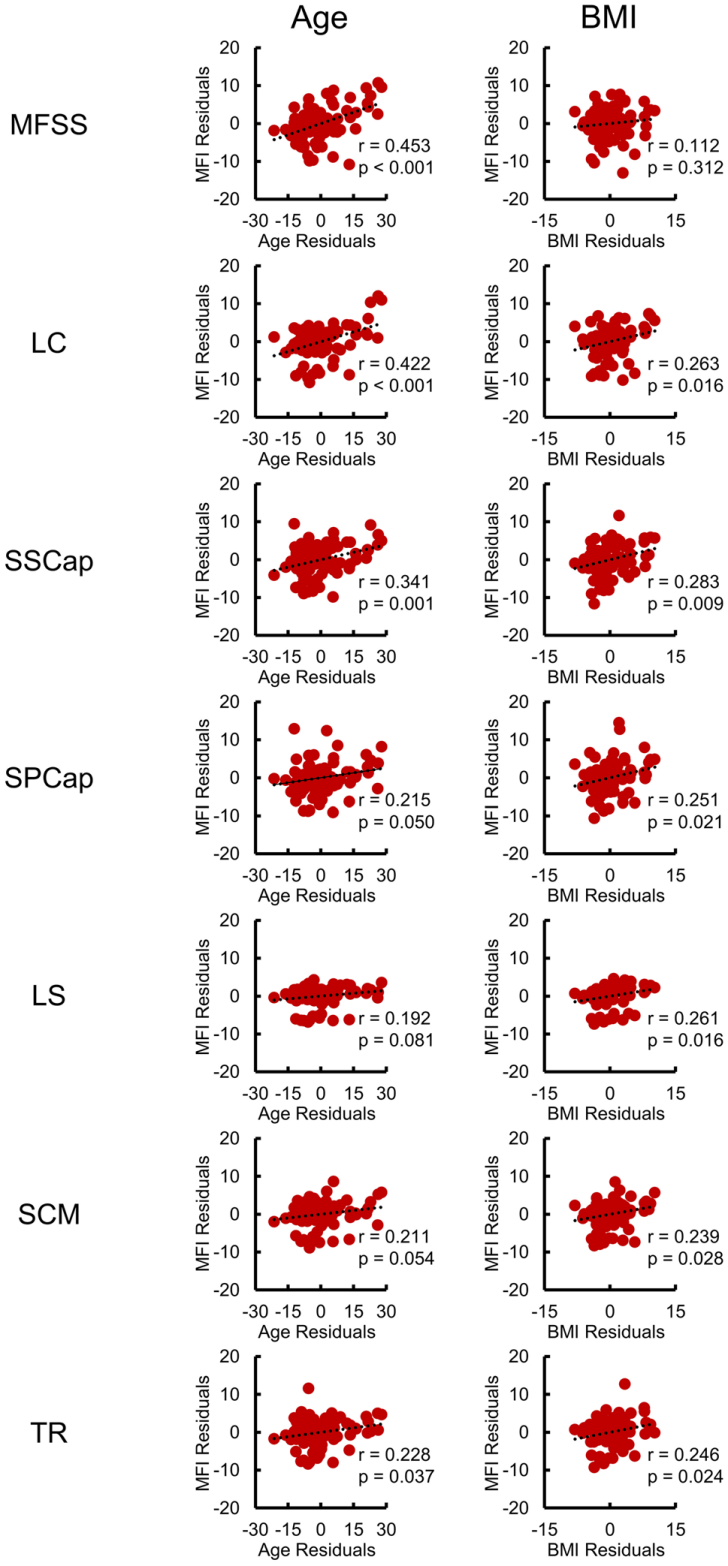
Relationship between muscle fat infiltration (MFI), age, and body mass index (BMI) across the muscle groups. Partial correlations (Pearson’s r) were performed to identify linear relationships between MFI and age or MFI and BMI in 84 participants from the first time point (< 2 weeks following a motor vehicle collision, 61 females, 23 males, age = 34.2 ± 10.7 years) after controlling for sex and BMI or sex and age, respectively. The left and right MFI measures for each muscle group were averaged for this analysis. For MFI and age, the residuals of MFI are plotted on the residuals of age after controlling for sex and BMI. For MFI and BMI, the residuals of MFI are plotted on the residuals of BMI after controlling for sex and age. Moderate correlations (r > 0.300) were only present between MFI and age for the deep cervical muscles (MFSS, LC, and SSCap).

Next, we assessed MFI between the muscle groups and the relationship of MFI to sex, age, and BMI in participants within two weeks of experiencing a whiplash injury from a MVC (See Supplementary Table 3 for sample characteristics). A repeated measures ANCOVA controlling for BMI and age demonstrated a significant within subject effect of the muscle group on MFI (F_(4.5,361.8)_ = 2.791, *p* = 0.021) and a between-subject effect of sex on MFI (F_(1,80)_ = 5.160, *p* = 0.026). MFI between each muscle group was positively correlated (Supplementary Fig. 4). Paired t-tests demonstrated significant differences in MFI between each muscle group with the deep cervical muscles (MFSS, LC, and SSCap) having greater MFI than the more superficial muscles (Fig. 5A). When averaging across all muscle groups, female participants had significantly higher MFI than male participants (1.8% ± 0.8% higher MFI in females, *p* = 0.026). Sex differences within each muscle groups were assessed with a one-way ANCOVA controlling for age and BMI. Females had significantly higher MFI than males in the SSCap, SPCap, and TR (Supplementary Table 3 and Fig. 5B). Two-tailed partial correlations controlling for sex and BMI demonstrated that age and MFI were moderately correlated (r > 0.300) in the deep cervical muscles (MFSS, LC, SSCap), while age and MFI were only weakly correlated (r < 0.300) in the more superficial muscles. Finally, BMI was only weakly correlated (r < 0.300) with MFI across the muscle groups after controlling for sex and age (Fig. 6).

## Discussion

We trained and tested a CNN model for segmentation of seven cervical spine muscle groups (left and right muscles segmented separately) using high-resolution fat-water Dixon MRI in participants within 2 weeks of a MVC-related whiplash injury. We demonstrate the feasibility of developing a CNN model for a complex, multi-muscle segmentation task: 14 muscles total. The trained CNN model was highly efficient – segmenting an image, and all muscles within that image, in less than 30 s compared to 4 to 8 h with manual segmentation. Across all muscle groups, we report high accuracy (mean bias < 2.0%) and high reliability (ICC_2__,__1_ > 0.800) for the CNN MFI measures compared to manual segmentation. Using the CNN measures, we identified higher MFI in females than males, and we also identified significant positive correlations between age (controlling for sex and BMI) and MFI, which was most pronounced (r > 0.300) for the deep cervical muscles: MFSS, LC, and SSCap. Overall, the CNN model permits the automatic extraction of accurate and reliable measures of muscle composition. The use of these measures as secondary markers in clinical trials, may lead to an improved understanding of spinal disease, delivering more sensitive and specific measures of spinal pathology, where the slow progression, often masked by age-related changes, pose a major roadblock to measuring therapeutic success. Such work could also advance the management of spinal conditions by encouraging efficiencies and innovation in clinical assessment and therapy development because the measures may detect change earlier than clinical endpoints (more sensitive) and are independent of assessor variability (more objective). Furthermore, these measures may aid in augmented intelligence-driven clinical decision making, allowing the clinician to better risk- and treatment-stratify patients using this information along with other clinical markers and endpoints^14^.

In the cervical spine, MFI has been reported to be present in greater magnitude in the deeper muscles surrounding the vertebrae, compared to the more superficial muscles^18,20^. We also found this to be true for our CNN MFI measurements supporting these previous findings. Prior research as well as our current study highlight the difficulty in determining whether elevated MFI is due to a pathological process or more part of normal aging, as age and MFI have been demonstrated to be associated^29,32–34^. For this study, the imaging data were from a parent study of individuals with neck pain due to a whiplash injury from a MVC, and thus aging as well as musculoskeletal trauma may have contributed to the MFI expressions in our cohort. We demonstrated higher MFI in females than males, which is consistent with previous findings in the lumbar spine musculature in asymptomatic^33^ and symptomatic^30^ participants. The effect of sex was present even when controlling for age and BMI. BMI, however, is only a rough estimate of body composition, and more accurate measures of body fat percentage (e.g., skinfold testing or bioimpedance) and physical function could help better understand the relationship between sex and MFI^35^. Sex and age are potential confounds when studying MFI. The inclusion of a sufficient number of male and female participants without whiplash symptoms following a MVC or an uninjured pain-free cohort is necessary to determine the nature of MFI expression and whether the magnitude is influenced by disorder severity, age, or sex.

While the accuracy and reliability of the CNN MFI measures were high for all muscle groups, the accuracy and reliability of the muscle volumes were generally lower, especially for the SPCap and LC, which had fair to poor reliability, respectively. The lower reliability of the muscle volumes was not unexpected, and several important limitations regarding the volume measurements should be noted. First, we chose not to segment the full length of each muscle group, and instead, limited the segmentations to the axial slices that cross the vertebral levels listed in Table 1 for each muscle group. This was to standardize the segmentation process because some muscles become difficult to differentiate close to their attachments and some muscles originated from outside the field of view (e.g. trapezius). Second, the axial slices do not cross most muscles perpendicularly, meaning that the area on an axial slice may not represent a true cross-sectional area of the muscle at that level. This is a common limitation for imaging studies that report cross-sectional area and is a good argument for reporting full volume instead, if possible^23^. While the CNN model only trained on the segmentations restricted to the pre-determined vertebral levels, the CNN model output was not restricted to these vertebral levels, and additional slices not included in the GT segmentations appear to have been included in the segmentations output by the CNN model. This is demonstrated by the volume ratio being greater than one for all muscle groups and a positive mean bias for muscle volume for all muscles except the TR, indicating that the CNN muscle segmentations produced a systematically greater volume than the GT. Despite the low CNN reliability for the volume measures, the mean bias in the muscle segmentations was less than 5 ml for all muscle groups, which is relatively low.

Despite advancements in MRI technology (e.g., improved image resolution and contrast), manual segmentation of the cervical spine muscles requires a high-level of knowledge in cervical spine anatomy and a substantial amount of time (i.e., 4 to 8 h per scan in this study). Due to the high-level of expertise and time requirements, we only trained and tested the CNN model using segmentations from one rater, which were then reviewed by an additional independent rater. This raises concern that the CNN model may be biased to a single rater’s interpretation of the anatomy. To mitigate this concern, in a subset of the dataset, we assessed the interrater reliability of manual segmentation with a third human rater, and we demonstrated excellent to good reliability for the MFI and volume measures of all muscles groups besides the TR muscle volume measures, which had poor reliability. Factors contributing to differences in segmentations between raters include the complex three-dimensional muscle and bone architecture as well as challenges in visualizing borders between adjacent muscle groups. The complexity of the TR muscular architecture likely led to the poor interrater reliability values for the TR muscle volume. The upper TR muscle fibers are primarily vertical near its cranial attachment, but fan out at the lower vertical levels, becoming almost parallel to the axial slices. Despite receiving similar instruction and training, the two raters may have used slightly different techniques to segment this muscle^23^. Caution should be used when interpreting the TR muscle measures as the segmentations likely do not generalize to all raters or participants.

The challenge of differentiating between adjacent muscles drove our previous decision to combine the multifidus and semispinalis cervicis (MFSS)^23^. The MFSS are both deep extensor muscles with similar actions. These muscle groups have many vertebral attachments and multiple layered fiber bundles that make boundaries challenging to differentiate. Similar reasoning was used for the grouping the longus colli and longus capitis (LC). In comparison to our previous CNN model for the MFSS, which used two-dimensional convolutional layers, here three-dimensional convolutional layers were employed. The choice of the three-dimensional model was due to the increased complexity of the muscle architecture, particularly of the superficial muscles, where the shape and spatial relationship of the muscles varies substantially along the superior-inferior axis. The three-dimensional model includes this additional spatial information, possibly allowing the model to better learn the complex three-dimensional architecture of the muscles, leading to improved performance.

In our previous CNN model, we trained the model using just the water-only images and limited the measure to the MFSS^24^. Here, we chose to train on the in-phase and out-of-phase images for two reasons. First, using the corresponding pairs of images generated from the Dixon sequence (e.g., fat-only and water-only images or in-phase and out-of-phase images), provides complementary information, which may have helped better define the muscle boundaries^36^. Second, the in-phase and out-of-phase images were used over the water-only and fat-only images due to the presence of the fat-water swapping artifact, which appears frequently in Dixon imaging (≈10% of the fat-water imaging scans)^37,38^. The fat-water swapping artifact results from misclassification of the fat and water signal in areas of magnetic field inhomogeneities, resulting in regions where fat-only and water-only images contain water-only and fat-only voxels, respectively. This artifact is not present in the in-phase and out-of-phase magnitude images. The use of both image contrasts does increase the number of features leading to a larger network size, higher model complexity, and increased computational costs. While we did not test whether the use of both the in-phase and out-of-phase images improves the segmentation performance, it is plausible as the images contain unique tissue contrast. Outputting the in-phase and out-of-phase images is an option on most scanners but may not be the default setting. With three-dimensional models, especially multi-modal models, memory does become an issue, limiting the batch size for training to only three datasets in this study. We are currently exploring the trade-off between the spatial window size and model performance to reduce the memory demands without sacrificing accuracy.

Here we employed the dense V-Net, a fully CNN model, to perform multi-muscle segmentation. We chose the dense V-Net because this model demonstrated state-of-the-art performance in a multi-organ segmentation task using abdominal CT images^39^. U-Net is another commonly used fully CNN segmentation model with both two-dimensional and three-dimensional architectures and structural similarities to V-Net^40,41^. U-Net has demonstrated high performance for lung segmentation from radiographs and bone segmentation from MRI in addition to many other tasks^42,43^. In this study, we did not compare different CNN architectures, but the results of a recent segmentation challenge showed similar performance between three-dimensional V-Net and three-dimensional U-Net models for a knee cartilage MRI segmentation task^44^. The application of CNN’s and deep learning into medical imaging analysis has been a major advancement in the field, leading to significant gains in segmentation performance across multiple medical imaging applications (for a comprehensive review see Hesamian et al. (2019))^45^. New architectures are continuing to be developed, leading to further improvements in segmentation performance over the V-Net and U-Net architectures^46^. Examples include recurrent neural networks, such as long short-term memory networks, and ensembles of neural networks^47,48^. Adopting these more advanced networks would likely improve the accuracy and reliability of the cervical spine muscle segmentations reported in this study as well as the resulting MFI measures.

We present these findings from the perspective of cervical spine conditions; however, we are actively working to expand this technology to the entire musculoskeletal system. A major barrier in developing the CNN models is the availability of large, diverse annotated datasets for training. The use of images from the same site, sequence, and imaging parameters likely reduces the generalizability of the trained CNN model and is a recognized limitation in this study. We are currently building a global coalition of musculoskeletal clinicians and researchers to pool clinical- and research-based imaging datasets to develop a large multi-site and multi-cultural annotated musculoskeletal imaging database for research purposes. Using this database, we aim to develop models that generalize to images of varying resolution, field-of-view, orientation, and image contrast (multi-modality and multi-scale) to establish normative reference values to inform clinical care on a patient-by-patient basis^49,50^. Fortunately, many past examples of open imaging databases for organizing and sharing imaging datasets exist to guide this process (e.g., OpenNeuro)^51^. Efficiently generating the annotated datasets with sufficient accuracy remains the greatest hurdle. We are currently exploring ways to employ crowd sourcing strategies and gamify the segmentation task, with the goal of developing a web-based educational platform targeted to health professional trainees to learn musculoskeletal anatomy interactively.

Based on our findings, we now have the technology to automate the segmentation of multiple muscles of the cervical spine, permitting the quantitative comprehensive assessment of cervical spine muscle composition. Our success in the cervical spine, with its architecturally complex anatomy, suggests that effectively extending these methods to other body regions is possible, and we have efforts underway in the lumbar spine, foot, leg, hip, and shoulder using both CT and MRI. The integration of the CNN models into the conventional clinical workflow as a postprocessing step should be straightforward, and in the not-too-distant future, these methods could provide clinicians with quantitative metrics of muscle characteristics extracted from the images obtained in a conventional musculoskeletal imaging series. Finally, these muscle measures would complement the examination and standard imaging findings and may provide increased diagnostic, prognostic, and predictive information to better inform the assessment and management of a wide variety of musculoskeletal and neuromuscular conditions. Relating these findings to clinical examinations across a patient population with varying levels of pain and disability is required before definitive conclusions can be made. This is well underway.

## Methods

### Participants

MRI datasets from 84 participants (61 females, 23 males, age = 34.2 ± 10.7 years) were obtained from a prospective observational longitudinal study exploring recovery from whiplash (ClinicalTrials.gov Identifier: NCT02157038). Datasets from the first (< 2 weeks post MVC) and fourth (12 months post MVC) study time points were used in the present study. Inclusion criteria included age 18 to 65 years, Quebec Task Force whiplash grades of II to III, and < 1 week post MVC with a primary complaint of neck pain. Exclusion criteria included a history of a previous MVC, spinal fracture, previous spinal surgery, previous diagnosis of cervical or lumbar radiculopathy, history of neurological or metabolic disorders, and contraindications to MRI. The study was approved by Northwestern University’s Institutional Review Board. All applicable institutional and governmental regulations concerning the ethical use of human volunteers were followed during the course of this research according to the Declaration of Helsinki, and written informed consent was obtained from every participant. Prior to working with the datasets, identifying personal information was removed.

### Image acquisition

A 3.0 T Siemens (Munich, Germany) Prisma scanner equipped with a 64-channel head/neck coil was used to acquire high-resolution three-dimensional fat-water images of the cervical and upper thoracic spine with a dual-echo gradient-echo FLASH sequence (2-point Dixon, TR = 7.05 ms, TE_1_ = 2.46 ms, TE_2_ = 3.69 ms, flip angle = 12°, bandwidth = 510 Hz/pixel, field-of-view = 190 mm × 320 mm, slab oversampling of 20% with 40 partitions to prevent aliasing in the anterior-posterior direction, in-plane resolution = 0.7 mm × 0.7 mm, slice thickness = 3.0 mm, number of averages = 6, acquisition time = 4 min 5 s)^52^. Fat and water have different chemical structures and precessional frequencies that differentially influence the local magnetic field. Images can be acquired when the fat and water signals are in-phase (IP = W + F) and out-of-phase (OOP = W – F). The in-phase and out-of-phase images can be combined to create images with fat-only signal (F = (IP – OOP) / 2) and water-only signal (W = (IP + OOP) / 2). As the images are acquired simultaneously in the same sequence and space, the images require no coregistration.

### Muscle segmentation

The muscle groups of interest included the left and right multifidus and semispinalis cervicis (MFSS), longus colli and longus capitis (LC), semispinalis capitis (SSCap), splenius capitis (SPCap), levator scapula (LS), sternocleidomastoid (SCM), and trapezius (TR). Each muscle was segmented manually by tracing their muscle borders on consecutive axial slices using methods previously described^23^. The muscle groups were segmented with separate labels for the left and right muscles at predetermined cervical levels where each muscle group is consistently present and can be accurately segmented. A mid-sagittal slice was used to identify the axial slices corresponding to each vertebral level (Table 1). The segmentation masks contained the background (label = 0) and each muscle labeled with an integer value (labels = 1–14). A single rater (VB) blinded to any demographic or clinical information segmented the 14 muscles of interest from 52 cervical spine Dixon scans. The segmentations were then reviewed by an additional independent, blinded rater (KW). Time required to segment a single Dixon scan ranged from 4 to 8 h. These segmentations were used as the ground truth (GT) for training and testing the CNN model. To assess interrater reliability, a third independent rater (RA), segmented a randomly selected subset of the Dixon scans (n = 10). All raters were doctoral level health professionals (physical therapy (RA) and chiropractic (VB and KW)) with extensive training in cervical spine anatomy and musculoskeletal imaging. The raters were permitted to use the fat, water, in-phase, and out-of-phase images to guide the segmentations.

### Data augmentation

Data augmentation was used to increase the variability in the training dataset. First, the images were split into training and testing datasets. The training dataset consisted of images from 17 participants (14 females, 3 males, age = 33.7 ± 11.4 years) with 17 scans from the first study time point and 17 scans from the fourth study time point (34 training scans total). The testing dataset consisted of images from 18 participants (11 females, 7 males, age = 31.7 ± 9.6 years) with 9 scans from the first time point and 9 scans from the fourth time point (18 testing scans total). Participants in the testing dataset were independent from the participants in the training dataset. From the training dataset, 2,000 augmented images were generated by applying a series of random mirroring (left-right flip), elastic deformation (number of control points = 3, sigma = 10), anisotropic spatial scaling (percentage =  ± 2.5), and rotation (left-right axis rotation =  ± 2.5°, anterior-posterior axis rotation =  ± 2.5°, superior-inferior axis rotation =  ± 5.0°). The non-augmented (i.e., raw) training images (n = 34) were used to assess the model performance across the training iterations using the Sørensen-Dice index (i.e., validation dataset). Data augmentation and training and testing the CNN model were performed using NiftyNet (Version 0.6.0), an open-source deep-learning platform built on TensorFlow (Version 1.15) in Python (Version 3.6) and designed specifically for medical imaging analysis^53^.

### Dense V-Net

CNNs are a class of deep neural networks that preserve spatial information in the network architecture and can learn patterns within images. Here we used the dense V-Net, a fully CNN model designed for segmentation tasks^39^. The dense V-Net consists of batch-wise spatial dropout, dense features stacks, V-Net downsampling and upsampling subnetworks, and dilated convolutions^54–56^. Skip connections in the V-Net architecture forward higher resolution information to the final segmentation. To limit bias towards predicting the image background, a loss function based on the Sørensen-Dice index (Dice Hinge) was employed and minimized. The output after soft-max transformation is probabilistic segmentation masks for each muscle with the same dimensions as the input volume.

### Training

Each dataset was first resampled to 0.7 mm × 0.7 mm × 3.0 mm resolution and zero padded (90 × 60 × 8 voxels). A three-dimensional dense V-Net model was trained using the in-phase and out-of-phase images of the augmented training dataset (spatial window = 360 × 240 × 32 voxels, learning rate = 0.001, activation function = ReLu, optimizer = Adam, loss function = Dice Hinge, regularization = ℓ2, decay = 0.00001, samples per volume = 3, batch size = 3, window sampling = uniform). Prior to training, histogram standardization and label normalization were performed. The dense V-Net model was initialized with random weights, and training was completed once the Sørensen-Dice index plateaued on the validation dataset.

### Performance

Performance of the CNN model was assessed using the Sørensen-Dice index (Dice), Jaccard index (JI), conformity coefficient (CC), true positive rate (TPR), true negative rate (TNR), positive predictive value (PPV), and volume ratio (VR) measures (Supplementary Table 1)^57^. Percent muscle fat infiltration (MFI) and muscle volume (ml) were measured using the segmentation masks from the GT and the CNN model for each muscle. MFI was calculated as the mean fat-only signal within a muscle divided by the sum of the mean fat-only signal and the mean water-only signal within a muscle multiplied by 100:

$$MFI = \frac{Fat}{{\left( {Fat + Water} \right)}} \times 100$$

Accuracy and reliability between the GT and the CNN model were assessed using Bland-Altman plots, Pearson correlations, and intraclass correlation coefficients (ICC_2,1_). The reliability between the two manual raters was also similarly calculated to assess interrater reliability between two human raters. For reliability and accuracy, MFI was considered the primary measure, while volume was used as a secondary measure to further assess the CNN segmentations.

### MFI assessment and characterization

Next, we used the trained CNN model to automatically segment the datasets of 84 participants from the first study time point (< 2 weeks following MVC, 61 females, 23 males, age = 34.2 ± 10.7 years) to examine differences in MFI between the muscle groups and then explore the relationship between MFI and sex, age, and BMI. The MFI of each muscle was calculated from the CNN segmentations, and then the left and right MFI measures for each muscle group were averaged to limit the number of comparisons and because we had no a priori hypotheses regarding left-right differences in MFI. A repeated measures ANCOVA with a within-subject variable of the muscle group, a between-subject factor of sex, and covariates of BMI and age was performed. This was followed by two-tailed paired t-tests to assess differences in MFI between the muscle groups, and pair-plots were generated to assess correlations in MFI between each muscle group. To identify sex differences in MFI for each muscle group, a one-way ANCOVA (i.e., multiple linear regression) was performed with a fixed factor of sex and covariates of age and BMI. Two-tailed partial Pearson correlations were performed to identify linear relationships between MFI and age or MFI and BMI while correcting for sex and BMI or sex and age, respectively. As these analyses were exploratory and aimed at further characterizing the MFI measures, no corrections for multiple comparisons were performed. Since the muscle groups were segmented at specific vertebral levels rather than across the entire superior-inferior extent of the muscle, the muscle volume measures obtained from the CNN model were not expected to represent an accurate measure of muscle size, and therefore, a more in-depth analysis of muscle volumes was not performed. Statistical analyses were performed using IBM SPSS Statistics (Version 27.0, Armonk, NY, USA), and an α < 0.05 was considered statistically significant.

## Supplementary Information

Supplementary Information.

## Data Availability

The de-identified datasets used in this study are available from the corresponding author upon reasonable request. The CNN segmentation model was developed using open-source Python packages (Tensorflow and NiftyNet). We are making the code and model openly available for transparency, replication, reproduction, and further research in more diverse samples. These will be made available on GitHub (https://github.com/kennethaweberii/). We will also release code to segment and compute volume and MFI from a cervical spine Dixon fat-water imaging scan.

## Abbreviations

CNN: Convolutional neural network
GT: Ground truth
MVC: Motor vehicle collision
MFI: Muscle fat infiltration
MFSS: Multifidus and semispinalis cervicis
LC: Longus colli and longus capitis
SSCap: Semispinalis capitis
SPCap: Splenius capitis
LS: Levator scapula
SCM: Sternocleidomastoid
TR: Trapezius
BMI: Body mass index
