# Supplementary material for: Multi-muscle deep learning segmentation to automate the quantification of muscle fat infiltration in cervical spine conditions
Source: Sci Rep. 2021 Aug 16;11:16567. doi: 10.1038/s41598-021-95972-x (PMC8368246; doi:10.1038/s41598-021-95972-x)
Supplement: Supplementary file 1 — Supplementary Information. [file 41598_2021_95972_MOESM1_ESM.pdf]

Multi-Muscle Deep Learning Segmentation to Automate the Quantification of Muscle Fat Infiltration in Cervical Spine Conditions

Kenneth A. Weber II, Rebecca Abbott, Vivie Bojilov, Andrew C. Smith, Marie Wasielewski, Trevor J. Hastie, Todd B. Parrish, Sean Mackey, and James M. Elliott

Supplementary Material

Supplementary Table 1. Segmentation Performance Metrics

Metric	Equation	Range	Meaning
Sørensen-Dice Index (DICE)	$\frac{2 \times \lvert SM \cap GT \rvert}{\lvert SM \rvert + \lvert GT \rvert}$	0 – 1	Spatial overlap between masks
Jaccard Index	$\frac{\lvert SM \cap GT \rvert}{\lvert SM \rvert + \lvert GT \rvert - \lvert SM \cap GT \rvert}$	0 – 1	Spatial overlap between masks
Conformity Coefficient	$1 - \frac{FP + FN}{TP}$	≤ 1	Ratio of incorrectly and correctly segmented voxels
True Positive Rate (TPR)	$\frac{TP}{TP + FN}$	0 – 1	Sensitivity
True Negative Rate (TNR)	$\frac{TN}{TN + FP}$	0 – 1	Specificity
Positive Predictive Value (PPV)	$\frac{TP}{TP + FP}$	0 – 1	Precision
Volume Ratio	$\frac{\lvert SM \rvert}{\lvert GT \rvert}$	≥ 0	Ratio of mask volumes

SM = segmentation mask; GT = ground truth mask; TP = true positive, voxels correctly segmented as muscle; TN = true negative, voxels correctly segmented as background; FP = false positive, voxels incorrectly segmented as muscle, FN = false negative, voxels incorrectly segmented as background.

Supplementary Table 2. Interrater Reliability

		MFI (%)			Volume (ml)		
**Muscle**		**$ICC_{2,1}$**	**95% CI**	**p**	**$ICC_{2,1}$**	**95% CI**	**p**
MFSS	Left	0.889	0.045 – 0.979	< 0.001	0.891	0.148 – 0.978	< 0.001
	Right	0.864	0.555 – 0.964	< 0.001	0.901	0.400 – 0.978	< 0.001
LC	Left	0.838	-0.048 – 0.970	< 0.001	0.670	0.148 – 0.905	0.012
	Right	0.827	-0.032 – 0.966	< 0.001	0.697	0.207 – 0.913	0.006
SSCap	Left	0.742	-0.078 – 0.946	< 0.001	0.927	0.419 – 0.985	< 0.001
	Right	0.847	-0.029 – 0.971	< 0.001	0.937	0.162 – 0.989	< 0.001
SPCap	Left	0.888	0.303 – 0.976	< 0.001	0.916	0.368 – 0.982	< 0.001
	Right	0.920	0.217 – 0.984	< 0.001	0.907	0.352 – 0.980	< 0.001
LS	Left	0.927	0.736 – 0.981	< 0.001	0.888	0.168 – 0.977	< 0.001
	Right	0.953	0.824 – 0.988	< 0.001	0.698	0.208 – 0.914	0.006
SCM	Left	0.936	0.549 – 0.986	< 0.001	0.952	0.822 – 0.988	< 0.001
	Right	0.947	0.274 – 0.990	< 0.001	0.942	0.795 – 0.985	< 0.001
TR	Left	0.924	0.736 – 0.980	< 0.001	0.118	-0.308 – 0.619	0.326
	Right	0.846	0.495 – 0.959	< 0.001	0.293	-0.152 – 0.724	0.088

The reliability between two manual raters was assessed in a randomly selected subset (n = 10) of the dataset using intraclass correlation coefficients ($ICC_{2,1}$, two-way random, absolute agreement, single measure) to evaluate the human-level interrater reliability. CI = confidence interval. p = F-test with true value = 0.

Supplementary Table 3. Baseline Imaging

Sample Characteristics

	Female (n = 61)	Male (n = 23)	t	p
Age, years	34.8 ± 10.9	32.7 ± 10.2	0.792	0.431
BMI, kg/m^2	25.2 ± 4.1	24.7 ± 3.6	0.502	0.617
NDI, %	38.1 ± 15.9	30.9 ± 19.2	1.756	0.083

MFI by Sex

Muscle	Female (n = 61)	Male (n = 23)	F	p
MFSS	16.4 ± 0.5	15.0 ± 0.9	1.825	0.181
LC	13.7 ± 0.5	13.1 ± 0.8	0.395	0.531
SSCap	15.5 ± 0.5	12.6 ± 0.8	10.263	**0.002**
SPCap	11.0 ± 0.5	7.5 ± 0.9	12.086	**0.001**
LS	7.5 ± 0.3	6.6 ± 0.56	1.773	0.187
SCM	9.7 ± 0.4	8.2 ± 0.7	3.492	0.065
TR	9.1 ± 0.4	7.3 ± 0.7	4.436	**0.038**

Sex differences in muscle fat infiltration (MFI) were explored using the datasets from the first study time point (< 2 weeks following motor vehicle collision, n = 84). Females and males did not statistically differ (independent samples t-test, equal variances assumed) by age, body mass index (BMI), or neck disability index (NDI). When averaging across all muscle groups, female participants had significantly higher MFI than male participants (1.8 % ± 0.8 % higher MFI in females, repeated measures ANCOVA controlling for BMI and age, $p = 0.026$). Results from a one-way ANCOVA controlling for age and BMI demonstrating significantly higher MFI for females than males in the SSCap, SPCap, and TR. MFI = Estimated marginal means ± 1 standard error. Bold = $p < 0.05$.

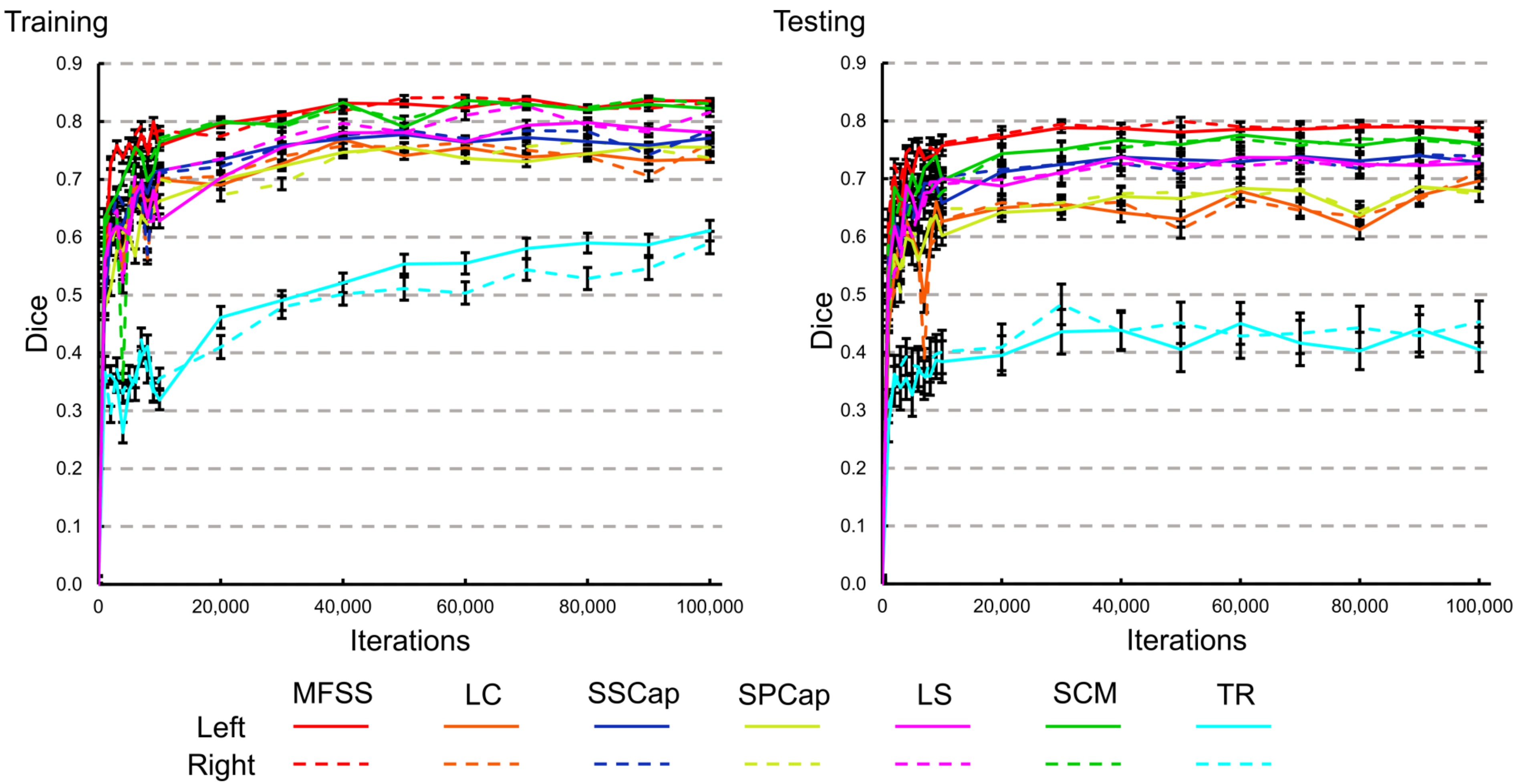

Supplementary Figure 1. Convolutional neural network (CNN) segmentation performance by muscle group across the training iterations. Segmentation performance across the training iterations on the training (n = 34) and testing datasets (n =18) was assessed using the Sørensen-Dice index (Dice). The non-augmented training datasets were used to assess the performance on the training dataset. Training of the three-dimensional CNN segmentation model was completed in 100,000 iterations. As expected, training performance is higher than the testing performance. Both the training and testing performance appears to plateau at 100,000 iterations without evidence of overfitting (i.e., reduction in testing performance). Error bars = 1 standard error.

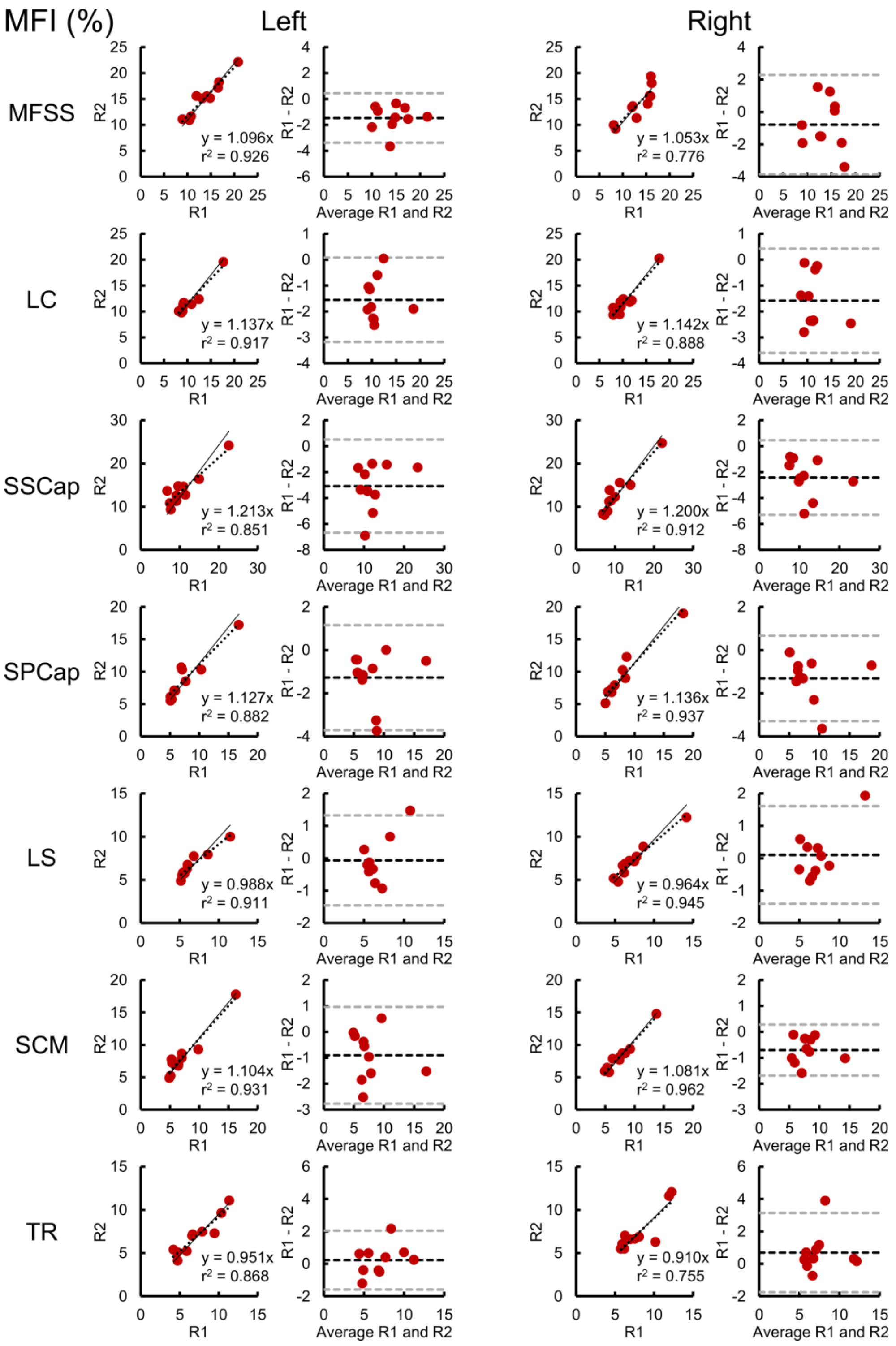

Supplementary Figure 2. Interrater reliability and accuracy for muscle fat infiltration measures (MFI) in a subset of the dataset (n = 10). Correlation and Bland-Altman plots are shown for MFI for each of the muscle groups. In the correlation plot, the dashed black line represents the best fit line, and the linear regression coefficient (β) of Rater 2 (R2) on Rater 1 (R1) (intercept = 0) is also provided (solid black line). In the Bland-Altman plots, the dashed black and gray lines indicate the mean difference (i.e., bias) ± 1.96 × standard deviation (i.e., 95% limits of agreement). See Supplementary Table 2 for intraclass correlation coefficients ($ICC_{2,1}$).

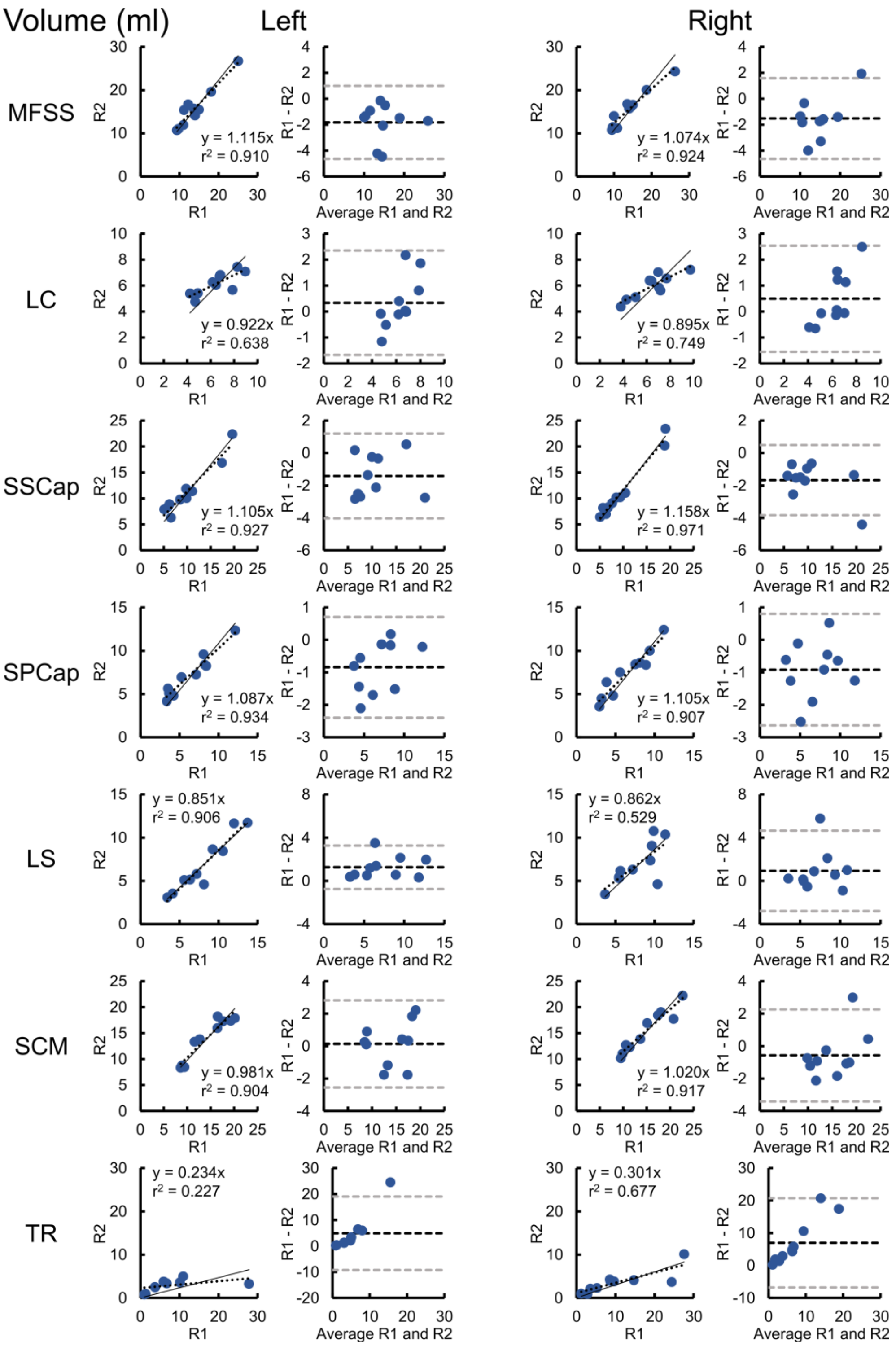

Supplementary Figure 3. Interrater reliability and accuracy of the convolutional neural network (CNN) muscle volumes in a subset of the dataset (n = 10). Correlation and Bland-Altman plots are shown for volume for each of the muscle groups. In the correlation plot, the dashed black line represents the best fit line, and the linear regression coefficient (β) of Rater 2 (R2) on Rater 1 (R1) (intercept = 0) is also provided (solid black line). In the Bland-Altman plots, the dashed black and gray lines indicate the mean difference (i.e., bias) ± 1.96 × standard deviation (i.e., 95% limits of agreement). See Supplementary Table 2 for intraclass correlation coefficients ($ICC_{2,1}$).

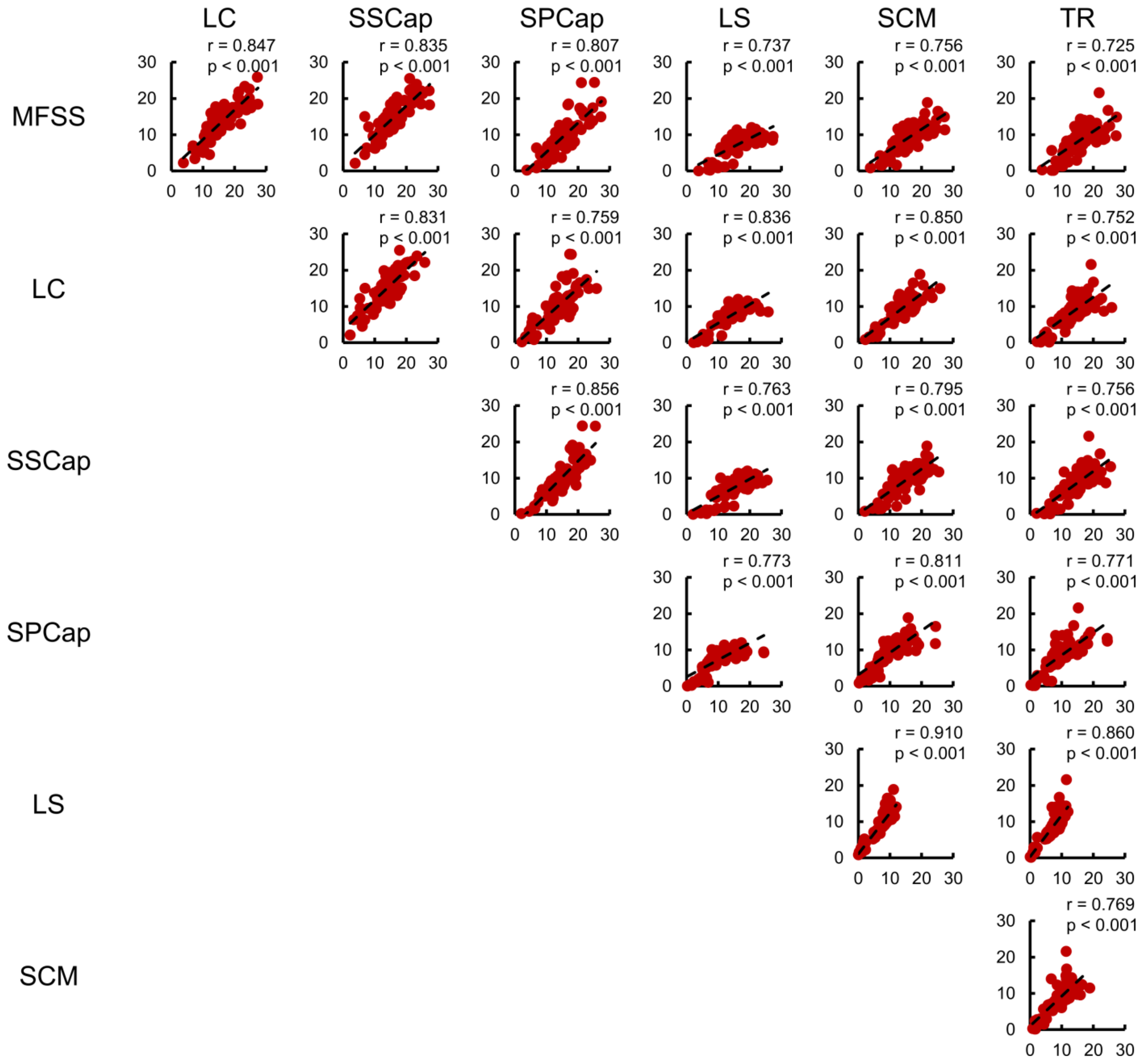

Supplementary Figure 4. Correlation of muscle fat infiltration (MFI) between muscle groups. The MFI of each muscle was calculated from the convolutional neural network (CNN) segmentations in 84 participants from the first time point (< 2 weeks following MVC, 61 females, 23 males, age = 34.2 ± 10.7 years). The left and right MFI measures for each muscle group were averaged for this analysis. Correlation pairs plots with Pearson correlation coefficients (r) are shown. The dashed black line represents the best fit line. The x-axis for each plot is represented by the muscle group specified in the left column, and the y-axis for each plot is represented by the muscle group specified in the top row.
